# Tension offloading improves cutaneous scar formation in Achilles tendon repair

**DOI:** 10.1093/jscr/rjac066

**Published:** 2022-03-09

**Authors:** Darren B Abbas, Hendrik Lintel, Michelle Griffin, Nicholas J Guardino, Jason L Guo, Amanda F Spielman, Asha C Cotterell, Jennifer B L Parker, Michael Januszyk, Derrick C Wan

**Affiliations:** Hagey Laboratory for Pediatric Regenerative Medicine, Stanford University School of Medicine, Stanford, CA, USA; Hagey Laboratory for Pediatric Regenerative Medicine, Stanford University School of Medicine, Stanford, CA, USA; Hagey Laboratory for Pediatric Regenerative Medicine, Stanford University School of Medicine, Stanford, CA, USA; Hagey Laboratory for Pediatric Regenerative Medicine, Stanford University School of Medicine, Stanford, CA, USA; Hagey Laboratory for Pediatric Regenerative Medicine, Stanford University School of Medicine, Stanford, CA, USA; Hagey Laboratory for Pediatric Regenerative Medicine, Stanford University School of Medicine, Stanford, CA, USA; Hagey Laboratory for Pediatric Regenerative Medicine, Stanford University School of Medicine, Stanford, CA, USA; Hagey Laboratory for Pediatric Regenerative Medicine, Stanford University School of Medicine, Stanford, CA, USA; Hagey Laboratory for Pediatric Regenerative Medicine, Stanford University School of Medicine, Stanford, CA, USA; Hagey Laboratory for Pediatric Regenerative Medicine, Stanford University School of Medicine, Stanford, CA, USA

**Keywords:** Achilles tendon, scar, hypertrophic scar, wound healing, embrace, tension offloading

## Abstract

Hypertrophic scar formation and non-healing wounds following Achilles tendon repair arise from poor vascularity to the incisional site or from excess mechanical stress/strain to the incision during the healing process. The embrace® scar therapy dressing is a tension offloading device for incisional scars. This study explored the effects of tension offloading during Achilles scar formation. A healthy 30-year-old male without any medical co-morbidities developed an acute rupture of his left Achilles tendon. The patient underwent open repair 1 week after injury. At post-operative day (POD) 14, the patient started daily tension offloading treatment on the inferior portion of the incision through POD 120. By POD 120, the untreated portion of the Achilles incision appeared hypertrophic and hyperpigmented, while the treated portion of the scar appeared flat with minimal pigmentation changes. The 12-week treatment of tension offloading on an Achilles tendon repair incision significantly improved cosmesis compared to untreated incision.

## INTRODUCTION

Achilles tendon ruptures occur at an incidence of 18 per 100 000 people annually [1]. Non-operative versus operative treatment is still controversial at this time, but surgery tends to be favored in the younger, athletic population [2]. However, as with any other surgical procedure, some negative outcomes may occur after operative repair, including wound infection, hypertrophic scar formation, scar adhesion, foreign body reaction, deep vein thrombosis, sural nerve injury, contracture and re-rupture [3–6]. These sequelae are multifactorial: native pathophysiology of scar formation, exogenous medication use, pre-existing medical conditions, operative technique, intraoperative complications, rehabilitation regimen, patient compliance and social determinants of health [7–10].

In the post-operative setting, most of these factors are out of the surgeon’s control unfortunately. However, the pathophysiology of a healing scar can still be manipulated in the post-operative setting by altering the biomechanical scaling forces acting upon a healing incision. The embrace® device is the only FDA-approved scar therapy system that is designed to relieve tension on a healing incision, leading to decreased wound healing complications [11, 12]. This device has been extensively studied in head and neck and abdominal scars but has never been utilized with Achilles tendon repair procedures. Therefore, with this study, we aimed to determine if this tension offloading device could improve skin scar formation following Achilles tendon repair.

## CASE REPORT

Disclosure of all protected health information within this study was approved by the Stanford University Institutional Review Board (IRB #33429). The patient was a healthy 30-year-old male who presented to Stanford University Sports Medicine Center for outpatient evaluation with a complaint of left ankle pain that occurred while playing basketball. The patient jumped up and, upon landing, immediately felt as though he had been kicked in the left ankle. The patient did not endorse any prodromal symptoms prior to this event, indicating potential Achilles tendinopathy. He also stated that he was unable to plantar flex his foot and, upon self-palpation, felt a significant divot in the area of his left Achilles tendon. He denied any recent antibiotic or steroid use, past medical or surgical history or smoking history.

**Figure 1:**
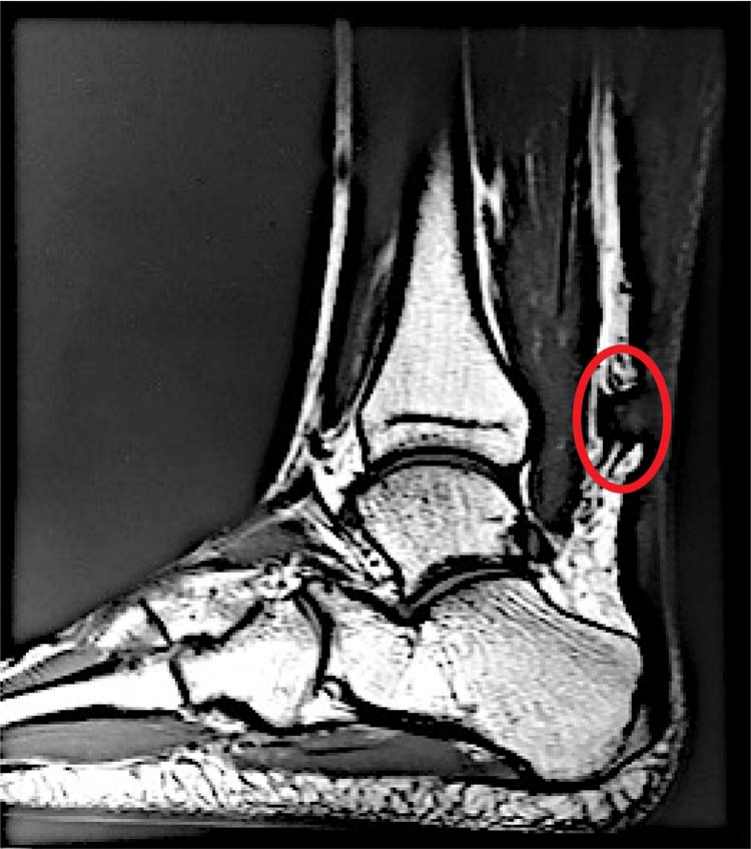
A sagittal view of the MRI of patient’s left Achilles demonstrates an acute, complete, Achilles rupture (circled in red) ~4 cm above the calcaneal insertion.

**Figure 2:**
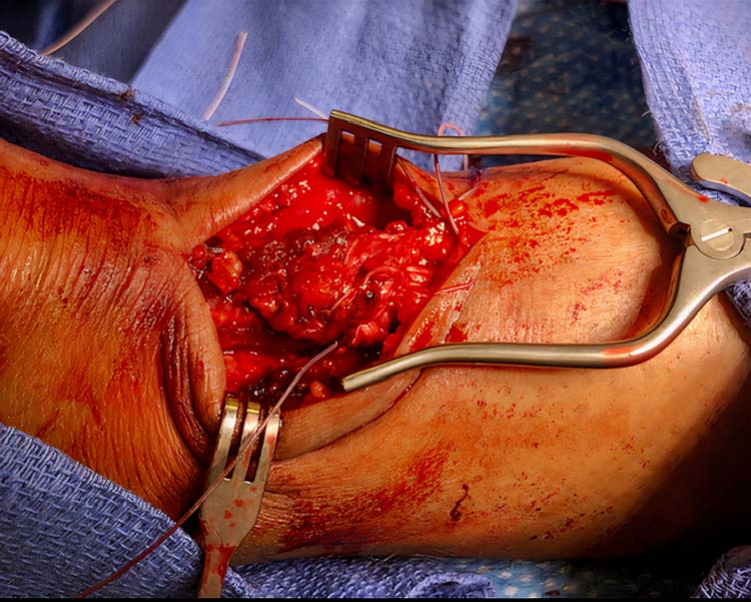
Intraoperative photograph demonstrating the open surgical technique utilized to repair the patient’s Achilles; initially, the proximal and distal stumps were debrided, controlled using Allis clamps and adequate excursion was noted with intraoperative testing; Krackow stitch was taken medially and laterally at the proximal stump and the distal stump with #2 FiberWire and the two edges of FiberWire were tied; #2 Orthocord was utilized to imbricate and reinforce with a Bunnell suture, followed by interrupted vicryl sutures; finally, the paratenon and subcutaneous tissue were closed in an interrupted fashion with vicryl sutures, while the epidermis was closed with interrupted nylon sutures.

**Figure 3:**
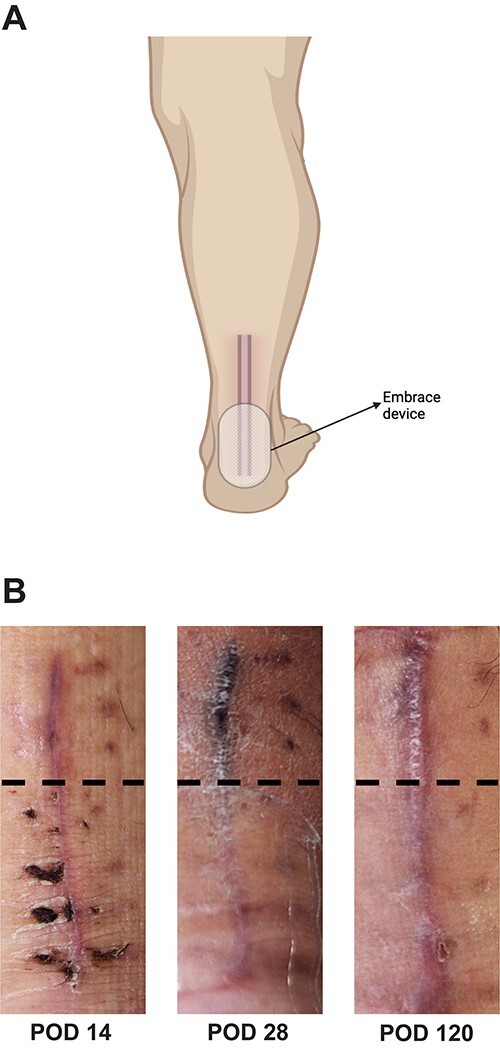
(**A**) Schematic representation of tension offloading device location and use during post-operative recovery from POD 14 until POD 120; (**B**) gross images of Achilles tendon repair incision prior to starting (POD 14), during (POD 28) and after completion (POD1 20) of treatment with the tension offloading device to the inferior portion of the incision; the middle panel (POD 28) demonstrates the patient actively utilizing the tension offloading device on the inferior portion of the incision; dotted black line demarcates non-treated scar (above the line) and treated scar (below the line).

On physical examination, a notable gap defect over the midsubstance of the left Achilles tendon was noted with swelling and edema of the left ankle. The patient also had loss of plantar flexion tone on the affected side as well as a positive Thompson test. He was neurovascularly intact with strong perfusion noted distally with palpable dorsalis pedis pulses. Radiographic confirmation with a magnetic resonance imaging (MRI) demonstrated a full-thickness tear of the left Achilles tendon 4 cm above the calcaneal insertion (Fig. 1). After extensive discussion regarding risks and benefits, a decision was made to undergo operative repair of this injury. Exactly 1-week post-injury, the patient underwent open primary repair of the left Achilles tendon without any complications (Fig. 2). In the immediate post-operative period, the patient was placed in a hard, immobile cast with plantar flexion that was then removed at post-operative day (POD) 14. After removal of the cast, the patient was started on an early functional rehabilitation program and in a functional brace for 6 weeks before transitioning to normal footwear. At POD 14, tension offloading therapy was begun on the inferior portion of the incision and was utilized daily until POD 120. The superior portion of the incision was left untreated for comparison. At POD 120, the tension offloading was discontinued (Fig. 3).

## DISCUSSION

Complications following operative repair of Achilles tendon rupture have not seen any significant decline in recent times. The location of the Achilles tendon makes the blood supply very tenuous and, as has been studied extensively in previous literature, a robust blood supply is one of the most important factors in the healing of a wound [13]. As blood supply is often dependent on each individual’s pre-existing conditions and cardiovascular health, this factor is often difficult to control for as a surgeon; however, in this patient’s case, there were no pre-existing medical conditions and strongly palpable distal pulses was appreciated, indicating good vascularity for wound healing. Given the initial appearance of the scar at POD 14, the incision appeared uniformly closed and well-healed. With no other foreseeable factors affecting wound healing and a well-compliant patient, this study then focused on altering the biochemical scale factors at play.

By decreasing tension on the incision, use of the tension offloading device during the post-operative period following Achilles tendon repair led to a significantly improved cosmesis of the treated scar. There was a significant improvement in pigmentation, width and height of the scar and overall appearance. This device was not only able to minimize these biomechanical scaling forces but decreased them to a point in which the scar grossly appeared better healed than the untreated portion of the incision.

Embrace® has been utilized and extensively studied in scar formation following breast reconstruction, head and neck surgery, abdominoplasty and thyroidectomy [11, 12]. Tension offloading for all these incisions demonstrated significant improvement in both cosmesis and functionality of the affected area. However, this case study is the first reported use of this tension offloading device for an Achilles tendon repair incision. This study shows that even in extremity scar formation, which notoriously have wound healing complications given their distal location from the heart, tension offloading can still play a significant role in prevention of these complications and improvement of scar formation. Furthermore, tension offloading of the skin following Achilles repair may mitigate mechanopathologic stimulus for scar formation associated with early post-operative mobilization and daily physical therapy range-of-motion exercises. Given the sudden increase in Achilles tendon ruptures since the onset of the COVID pandemic, this case study provides data of this device’s potential therapeutic use to the increasing number of patients undergoing this procedure [14, 15].

## CONCLUSION

This case report shows that tension relief on an Achilles tendon repair incision may improve wound healing and decrease scar pigmentation and hypertrophy. This is the first study displaying the power of tension offloading on the cutaneous portion of an Achilles tendon repair incision.
